# COVID-19 in Schleswig-Holstein: Infektionsepidemiologische Auswertungen von März bis September 2020

**DOI:** 10.1007/s00103-021-03301-4

**Published:** 2021-03-21

**Authors:** Ruben Rose, Damian Scherer, Gregor Maschkowitz, Christoph Läubrich, Helmut Fickenscher

**Affiliations:** 1 Kompetenzzentrum für das Meldewesen übertragbarer Krankheiten in Schleswig-Holstein, am Institut für Infektionsmedizin, Christian-Albrechts-Universität zu Kiel und Universitätsklinikum Schleswig-Holstein, Kiel, Deutschland; 2 Institut für Infektionsmedizin, Christian-Albrechts-Universität zu Kiel und Universitätsklinikum Schleswig-Holstein, Brunswiker Str. 4, 24105 Kiel, Deutschland

**Keywords:** Infektionen mit SARS-CoV‑2, COVID-19, Schleswig-Holstein, Meldepflicht, Ausbruchsmeldungen, SARS-CoV‑2 infections, COVID-19, Schleswig-Holstein, Reporting of notifiable diseases, Outbreak reporting

## Abstract

Die COVID-19-Pandemie stellt das deutsche Meldewesen im öffentlichen Infektionsschutz vor große Herausforderungen. Im Bundesland Schleswig-Holstein unterstützt die Landesmeldestelle die Gesundheitsämter durch tägliche und wöchentliche Auswertungen und hilft bei der Übermittlung der Meldedaten gemäß Infektionsschutzgesetz an das Robert Koch-Institut.

In dem vorliegenden Bericht der Landesmeldestelle Schleswig-Holstein werden die SARS-CoV-2-Meldedaten aus dem Zeitraum März bis September 2020 ausgewertet. In Orientierung an der Entwicklung der Infektionszahlen wurde der Zeitraum in zwei Phasen ähnlichen Umfangs eingeteilt: März bis Mai und Juni bis September. Insgesamt wurden 4898 Infektionsfälle gemeldet. Bei dem Vergleich der Phasen zeigten sich besonders deutliche Unterschiede hinsichtlich der Hospitalisierung und Letalität, des Alters und der Staaten des Infektionsorts. In der ersten Phase waren besonders ältere Personen von einer hohen Hospitalisierungsrate und Sterblichkeit betroffen. In der zweiten Phase lagen das durchschnittliche Alter und die Hospitalisierungs- und Sterberaten deutlich niedriger und ein besonders großer Anteil war mit internationaler Reiseaktivität verbunden. Die Auswertung der Ausbruchsdokumentationen ergab den besonderen Schwerpunkt im Setting der privaten Haushalte. Dieser Artikel beschreibt die epidemische Situation in einem im Bundesvergleich Niedriginzidenzland.

## Abstract (en)

The COVID-19 pandemic poses major challenges for the German notification system in public infection control. For the federal state of Schleswig-Holstein evaluations, the state reporting office supports the public health departments by providing daily and weekly evaluations and supports the transmission of notification data to the Robert Koch Institute according to the Infection Protection Act.

In the present report of the state notification office of Schleswig-Holstein, the SARS-CoV‑2 reporting data for the period from March to September 2020 are evaluated. Based on the development of the infection numbers, this period was divided into two phases of similar size: March to May and June to September. A total of 4898 infection cases were reported. Upon comparison of the phases, there were particularly marked differences in hospitalization and mortality, age, and countries of infection site. In the first phase, elderly persons were particularly affected by high rates of hospitalization and mortality. In the second phase, the average age and hospitalization and mortality rates were significantly lower, and a particularly large proportion were associated with international travel activity. The evaluation of the outbreak documentation revealed a particular focus in private household settings. This article describes the epidemic situation in a low-incidence state within the Federal Republic of Germany.

## Einleitung

Die COVID-19-Pandemie mit dem Erreger SARS-CoV‑2 (schweres akutes Atemwegssyndrom-Coronavirus-Typ 2) erfasste gegen Ende Februar 2020 Deutschland und damit auch das Bundesland Schleswig-Holstein. Die Meldedaten gemäß Infektionsschutzgesetz (IfSG) werden von den Gesundheitsämtern erfasst. In Schleswig-Holstein unterstützt die Landesmeldestelle die Gesundheitsämter durch tägliche und wöchentliche Auswertungen und hilft bei der Übermittlung der Meldedaten gemäß Infektionsschutzgesetz an das Robert Koch-Institut. Der weit überwiegende Teil der Meldungen wird täglich bis 20 Uhr übermittelt.

Die rechtliche Grundlage für die Erfassung der Meldedaten wurde am 30.01.2020 durch die „Verordnung über die Ausdehnung der Meldepflicht nach §6 Abs. 1 Satz 1 Nr. 1 und §7 Abs. 1 Satz 1 des Infektionsschutzgesetzes (IfSG) auf Infektionen mit dem neuartigen Coronavirus“ gelegt. Aufgrund der Falldefinitionen für die Coronavirus-Krankheit-2019 (COVID-19) des RKI vom 29.05.2020 wurden Erregernachweise, Erkrankungs- oder Todesfälle übermittelt [1]. Der labordiagnostische Nachweis der Ribonukleinsäure (RNA) von SARS-CoV‑2 ist Grundlage der Meldung und erfüllt die Referenzdefinition. Für die Meldung der Erkrankung sind als Kriterien definiert: das klinische Bild der Pneumonie, ein unspezifisches Bild mit akuter respiratorischer Symptomatik jeder Schwere oder der krankheitsbedingte Tod. In dieser Arbeit werden nur Fälle mit Erregernachweis und somit Erfüllung der Referenzdefinition nach dem Stand des 29.05.2020 untersucht.

Die namentliche Meldepflicht basiert auf §6 Abs. 1 Satz 1 Nr. 1 Buchst. t IfSG und schließt den Krankheitsverdacht, die Erkrankung sowie den Tod ein. Nach §7 Abs. 1 Nr. 44a IfSG umfasst die namentliche Meldepflicht den direkten Nachweis von SARS-CoV‑2, soweit er auf eine akute Infektion hinweist. Zu Beginn der Pandemie wurden Nachweise von SARS-CoV‑2 der allgemeinen Meldekategorie „Weitere bedrohliche Krankheiten“ (WBK) zugeordnet. Ab dem 17.04.2020 wurden sie im zentralen Meldeprogramm SurvNet der neuen Kategorie CVD (für COVID-19) zugewiesen. Eine kurze Übersicht folgt über die wichtigsten Ereignisse im Zusammenhang mit der COVID-19-Pandemie mit Fokus auf Schleswig-Holstein. Bereits am 01.12.2019 wurden erste COVID-19-Fälle in Wuhan in China beobachtet. Am 01.01.2020 wurde ein Markt für Fisch und Schlachttiere in Wuhan geschlossen. Am 13.01.2020 wurde der erste Erkrankungsfall außerhalb Chinas in Thailand berichtet. Die Abriegelung der Stadt Wuhan erfolgte am 23.01.2020. Die Weltgesundheitsorganisation (WHO) erklärte am 30.01.2020 die „gesundheitliche Notlage internationaler Tragweite“ und am 11.03.2020 den Pandemiefall. Nachdem am 27.02.2020 der erste Erkrankungsfall in Deutschland in der Region München gemeldet wurde, fand am 28.02.2020 die Meldung des ersten Erkrankungsfalls für Schleswig-Holstein statt. Der erste Todesfall eines Schleswig-Holsteiners in Ägypten wurde am 08.03.2020 berichtet und der erste Todesfall in Schleswig-Holstein am 17.03.2020. Am 18.03.2020 trat ein einschränkendes Maßnahmenpaket in Schleswig-Holstein in Kraft, das unter anderem die Schließung der Schulen und Kindergärten sowie der Restaurants, Besuchsverbote in Kliniken und die Untersagung öffentlicher Veranstaltungen einschloss. Über weitere Landesverordnungen folgten das Tourismusverbot in Schleswig-Holstein und wesentliche Kontaktbeschränkungen. Am 25.03.2020 erklärte der Bundestag die „epidemische Lage von nationaler Tragweite“ und verabschiedete am 27.03.2020 das erste „Gesetz zum Schutz der Bevölkerung bei epidemischer Lage von nationaler Tragweite“ sowie am 19.05.2020 das zweite Gesetz mit gleicher Bezeichnung. Seit dem 05.05.2020 wurden schrittweise Lockerungen der einschränkenden Maßnahmen wirksam. Die Landesverordnungen Schleswig-Holstein wurden mehrfach aktualisiert.

In diesem Bericht mit Datenstand zum 09.10.2020 wird die Epidemiologie der SARS-CoV-2-Pandemie in Schleswig-Holstein im Zeitraum März bis September 2020 beschrieben. Bereits vorliegende Auswertungen der Landesmeldestelle Schleswig-Holsteins zu der ersten Pandemiewelle werden damit fortgeschrieben [2].

## Auswertung der Meldedaten von März bis September 2020

Im Beobachtungszeitraum März bis September 2020 wurden in Schleswig-Holstein insgesamt 11.564 Fälle jeglicher meldepflichtiger Infektionskrankheiten gemeldet, von denen 9204 die jeweilige Referenzdefinition erfüllten. Unter allen Meldungen wurde bei 4898 Personen die RNA des SARS-CoV‑2 nachgewiesen. Davon wurden 611 Personen stationär behandelt (12,7 %) und 162 Todesfälle (3,4 %) wurden berichtet.

**Figure 1:**
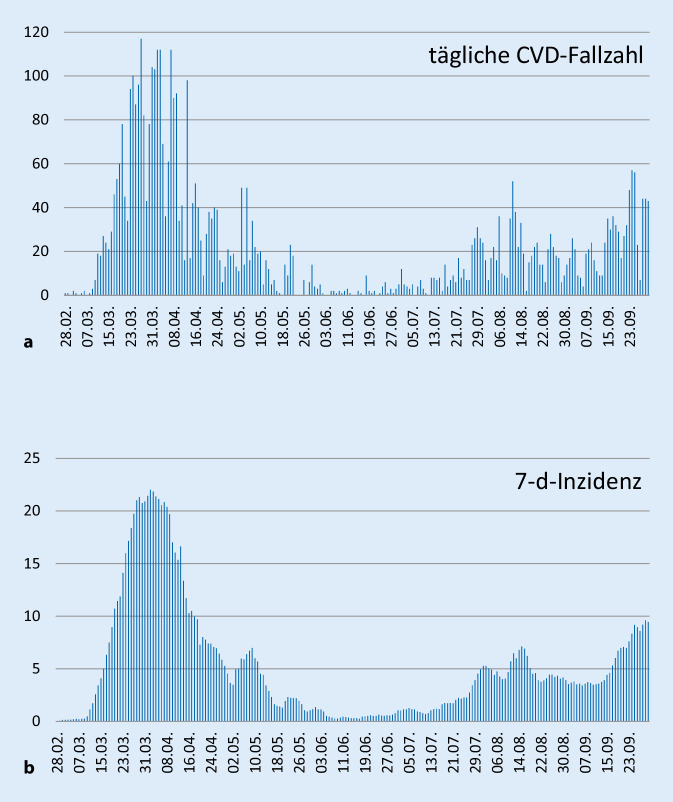

Anhand der Entwicklung des Infektionsgeschehens wurde der untersuchte Zeitraum in zwei epidemische Phasen eingeteilt: 1) März bis Mai und 2) Juni bis September 2020. Die Pandemie begann in deutlichem Umfang in Schleswig-Holstein Anfang März und erreichte ihr Maximum in der letzten Märzwoche. Nach der Einführung einschränkender Maßnahmen wurde die Ausbreitung zeitnah verlangsamt und schon Anfang Juni (Beginn der zweiten Phase) lag nur noch eine minimale Fallzahl vor. Im Monat Juli wurde ein deutlicher Anstieg der täglichen Fallzahlen und der 7‑Tage-Inzidenzen (pro 100.000 Einwohner) beobachtet, auf den dann in der zweiten Hälfte des Septembers ein weiterer Anstieg auf ca. die Hälfte der täglichen Fallzahlen und der 7‑Tage-Inzidenzen der ersten Phase folgte (Abb. 1a, b). Die zweite Phase setzte sich weit über den Jahreswechsel im Jahr 2021 fort, allerdings mit veränderten Schwerpunkten.

**Figure 2:**
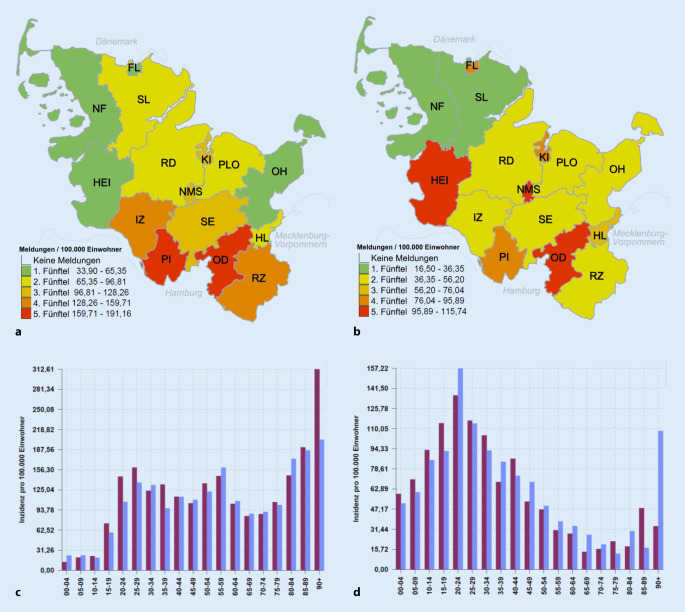

In der ersten Phase lag der Schwerpunkt der geografischen Verteilung in den Hamburg-nahen Kreisen Pinneberg und Stormarn; in der zweiten Phase wirkten sich unübersichtliche Ausbruchssituationen im Kreis Dithmarschen und in Neumünster zusätzlich stark aus (Abb. 2a, b). Die Altersgruppenverteilung der ersten Phase war durch niedrige Fallzahlen bei Kindern und Jugendlichen sowie durch deutlich erhöhte Fallzahlen bei älteren Personen geprägt. Im Gegensatz dazu betraf die zweite Phase hauptsächlich junge Erwachsene und vergleichsweise in geringem Maß ältere Personen (Abb. 2c, d).

Der jüngste Patient war ein Säugling, der älteste Patient war 119 Jahre alt. Ab dem 45. Lebensjahr stieg die Hospitalisierungsrate kontinuierlich an und ab dem 60. Lebensjahr steigerte sich die Letalität. Von den 163 verstorbenen infizierten Patienten wurde die Todesursache bei 146 Fällen der SARS-CoV-2-Infektion zugeschrieben, bei 17 Fällen wurde eine andere Todesursache festgestellt und in einem Fall fehlte diese Angabe. Insgesamt 120 der Verstorbenen waren im Rahmen der Erkrankung hospitalisiert.

Die Kernparameter wurden bei asymptomatischen, symptomatischen und hospitalisierten SARS-CoV-2-infizierten Personen und Verstorbenen vergleichend betrachtet (Tab. 1 und 2). Hierfür wurden alle 4827 Personen mit PCR-Nachweis des SARS-CoV‑2 und entsprechenden Angaben in vier Gruppen eingeteilt: 1. infizierte Personen ohne Symptomatik oder mit einer für COVID-19 nicht relevanten Symptomatik; 2. symptomatische Personen mit relevanter Symptomatik, aber ohne hospitalisierte oder verstorbene Personen; 3. hospitalisierte Personen, aber ohne Verstorbene; 4. verstorbene Personen ungeachtet einer vorherigen Hospitalisierung.

**Table 1:** 

Kreis	Einwohnerzahl^a^	Inzidenz^b^			Fallzahl			Hospitalisiert			Hospitalisierungsrate %			Verstorben			Letalität %		
Phase		1	2	1+2	1	2	1+2	1	2	1+2	1	2	1+2	1	2	1+2	1	2	1+2
Pinneberg (PI)	316.103	190	86	276	601	273	874	129	13	142	21,5	4,8	16,2	46	1	47	7,65	0,37	5,38
Stormarn (OD)	244.156	171	96	267	418	234	652	51	8	59	12,2	3,4	9,0	33	2	35	7,89	0,85	5,37
Neumünster (NMS)	80.196	97	113	211	78	91	169	9	4	13	11,5	4,4	7,7	2	1	3	2,56	1,10	1,78
Kiel (KI)	246.794	113	76	189	279	187	466	64	16	80	22,9	8,6	17,2	10	2	12	3,58	1,07	2,58
Herzogtum Lauenburg (RZ)	198.019	133	49	182	264	97	361	47	4	51	17,8	4,1	14,1	17	1	18	6,44	1,03	4,99
Steinburg (IZ)	131.013	136	37	173	178	48	226	22	3	25	12,4	6,3	11,1	3	0	3	1,69	0,00	1,33
Segeberg (SE)	277.175	118	51	169	326	142	468	43	4	47	13,2	2,8	10,0	7	0	7	2,15	0,00	1,50
Dithmarschen (HEI)	133.193	44	114	158	59	152	211	13	18	31	22,0	11,8	14,7	3	2	5	5,08	1,32	2,37
Lübeck (HL)	216.530	77	57	133	166	123	289	8	5	13	4,8	4,1	4,5	1	0	1	0,60	0,00	0,35
Rendsburg-Eckernförde (RD)	274.098	93	38	131	255	105	360	47	4	51	18,4	3,8	14,2	14	0	14	5,49	0,00	3,89
Plön (PLÖ)	128.686	93	37	130	120	47	167	27	9	36	22,5	19,1	21,6	8	0	8	6,67	0,00	4,79
Flensburg (FL)	90.164	47	82	129	42	74	116	6	2	8	14,3	2,7	6,9	3	0	3	7,14	0,00	2,59
Schleswig-Flensburg (SL)	201.156	79	15	94	158	31	189	18	3	21	11,4	9,7	11,1	4	0	4	2,53	0,00	2,12
Nordfriesland (NF)	165.951	53	30	83	88	50	138	13	4	17	14,8	8,0	12,3	1	1	2	1,14	2,00	1,45
Ostholstein (OH)	200.539	34	36	70	68	73	141	7	10	17	10,3	13,7	12,1	0	0	0	0,00	0,00	0,00
*Schleswig-Holstein*	*2.903.773*	*107*	*59*	*166*	*3100*	*1727*	*4827*	*504*	*107*	*611*	*16,3*	*6,2*	*12,7*	*152*	*10*	*162*	*4,90*	*0,58*	*3,36*

^a^Einwohnerzahl nach Destatis zum 31.12.2019

^b^ pro 100.000 Einwohner

**Table 2:** 

	Insgesamt			Asymptomatisch			Symptomatisch			Hospitalisiert			Verstorben		
Phase	1	2	1+2	1	2	1+2	1	2	1+2	1	2	1+2	1	2	1+2
*Geschlecht und Alter*															
Männlich, Anzahl	1462	874	2336	248	333	581	956	479	1435	206	50	256	93	4	97
Männlich, MW Alter	49,6	34,4	43,9	49,8	32,9	40,1	44,9	34,1	41,3	63,7	56,1	62,2	76,9	69,3	76,6
Weiblich, Anzahl	1638	853	2491	286	321	607	1147	476	1623	186	49	235	59	6	65
Weiblich, MW Alter	51,7	34,2	45,7	60,9	32,6	46,0	46,0	34,4	42,6	68,2	51,8	64,7	82,8	87,2	83,2
Gesamt, Anzahl	3100	1727	4827	534	654	1188	2103	955	3058	392	99	491	152	10	162
Gesamt, MW Alter	50,7	34,3	44,8	55,8	32,8	43,1	45,5	34,2	42,0	65,8	54,0	63,4	79,2	80,0	79,2
*Altersgruppen*															
0–9	50	150	200	7	91	98	40	49	89	3	5	8	0	0	0
10–19	124	264	388	20	125	145	98	124	222	6	3	9	0	0	0
20–29	438	408	846	67	124	191	355	266	621	17	9	26	0	0	0
30–39	402	276	678	55	90	145	332	173	505	14	13	27	0	0	0
40–49	402	242	644	74	78	152	301	144	445	23	16	39	3	0	3
50–59	689	195	884	76	62	138	555	122	677	61	10	71	6	0	6
60–69	343	84	427	50	33	83	218	46	264	73	8	81	16	2	18
70–79	293	51	344	67	21	88	114	18	132	93	14	107	42	3	45
80–89	279	39	318	82	21	103	75	9	84	89	17	106	60	3	63
90–99	72	13	85	31	9	40	14	1	15	12	4	16	23	1	24
100–119	8	2	10	5	0	5	1	1	2	1	0	1	2	1	3
*Klinik*															
Klinik vorhanden	1959	480	2439	0	0	0	1604	444	2048	245	29	274	109	3	112
Ja, relevant für COVID‑19	604	554	1158	0	0	0	499	511	1010	79	28	107	23	3	26
Ja, aber nicht relevant	142	219	361	142	219	361	0	0	0	29	20	49	5	3	8
Klinik nicht vorhanden	392	435	827	392	435	827	0	0	0	38	20	58	15	1	16
Nicht erhoben	3	39	42	0	0	0	0	0	0	1	2	3	0	0	0
*Symptomatik*															
Fieber	1149	386	1535	0	0	0	888	348	1236	197	31	228	62	2	64
Husten	1477	464	1941	1	1	2	1248	425	1673	185	27	212	40	3	43
Allgemeinsymptome	1272	584	1857	2	1	3	1040	532	1572	165	39	204	65	3	68
Schnupfen	693	361	1054	0	1	1	647	346	993	39	8	47	6	0	6
Halsschmerzen	549	390	939	0	1	1	506	365	871	36	18	54	6	1	7
Dyspnoe	266	36	302	0	0	0	137	20	157	83	11	94	46	3	49
Durchfall	200	65	265	1	1	2	162	56	218	32	8	40	6	0	6
Geschmacksveränderung^a^	123	223	346	0	0	0	115	207	322	8	13	21	0	0	0
Geruchssinnveränderung^a^	105	181	286	0	0	0	97	170	267	8	9	17	0	0	0
Pneumonie	64	9	73	0	0	0	1	1	2	30	5	35	33	3	36
Beatmung	31	4	35	0	0	0	2	2	4	10	0	10	19	2	21
Akutes Lungenversagen (ARDS)	29	9	38	0	0	0	1	6	7	12	2	14	16	1	17
Tachykardie	9	6	15	0	0	0	4	4	8	3	2	5	2	0	2
Tachypnoe	7	3	10	0	0	0	2	1	3	2	2	4	3	0	3
*Risikokonstellation*															
Herz-Kreislauf-Erkrankung	442	123	565	19	11	30	204	58	262	132	21	153	57	5	62
Nervensystemerkrankung	186	19	205	35	15	50	73	4	77	61	5	66	33	2	35
Lungenerkrankungen	149	56	205	18	18	36	81	32	113	44	6	50	14	2	16
Diabetes	120	48	168	23	19	42	46	23	69	44	7	51	17	2	19
Krebserkrankungen	106	24	130	23	11	34	44	10	54	37	7	44	17	0	17
Nierenerkrankungen	84	18	102	32	11	34	11	5	16	43	7	50	18	1	19
Immunschwäche	69	13	82	7	5	12	35	5	40	20	4	24	12	0	12
Lebererkrankungen	23	8	31	2	4	6	11	4	15	6	2	8	5	0	5
Schwangerschaft	10	11	21	1	2	3	8	8	16	1	1	2	0	0	0
Postpartalphase	3	2	5	0	1	1	2	1	3	1	0	1	0	0	0
*Status nach §§ 23, 33, 36, 42*^*b*^															
Betreut nach § 23	77	30	107	23	22	45	13	1	14	51	12	0	11	3	14
Betreut nach § 33	54	196	250	15	108	123	36	83	119	4	4	8	0	0	0
Betreut nach § 36	303	37	340	127	28	155	76	7	83	51	2	53	68	2	70
Tätig nach § 23	346	78	424	43	25	68	279	53	332	21	0	21	3	0	3
Tätig nach § 33	54	47	101	3	7	10	48	38	86	3	1	4	0	0	0
Tätig nach § 36	171	36	207	25	13	38	139	22	161	6	1	7	1	0	1
Tätig nach § 42	183	32	215	108	15	123	71	15	86	5	2	7	0	0	0
*Expositionsländer*															
Schleswig-Holstein (SH)	2027	909	1468	348	270	618	1305	555	1860	254	60	314	118	7	125
Hamburg (HH)	155	55	210	8	10	18	114	44	158	27	0	27	6	0	6
Deutschland, ohne SH und HH	111	52	163	5	10	15	85	36	121	18	5	23	3	0	3
Österreich	243	12	255	15	3	18	215	9	224	12	0	12	1	0	1
Mitteleuropa, weitere	9	14	23	1	2	3	6	12	18	1	0	1	1	0	1
Nordeuropa	10	14	24	2	0	2	7	14	21	0	0	0	1	0	1
Westeuropa	29	17	46	3	8	11	23	9	32	3	0	3	0	0	0
Südeuropa	62	70	132	5	34	39	52	35	87	3	1	4	2	0	2
Osteuropa	4	40	44	0	11	11	3	28	31	1	1	2	0	0	0
Südosteuropa	1	244	245	1	155	156	0	77	77	0	1	1	0	0	0
Europa, weitere	3	0	3	0	0	0	3	0	3	0	0	0	0	0	0
Türkei	4	93	97	0	50	50	3	38	41	1	0	1	0	1	1
Asien, weitere	13	16	29	3	10	13	8	3	11	2	3	5	0	0	0
Amerika	30	2	32	0	1	1	27	1	28	3	0	3	0	0	0
Afrika	21	8	29	1	6	7	17	1	18	1	1	2	2	0	2
Australien	4	0	4	0	0	0	2	0	2	2	0	2	0	0	0
Ausland, undifferenziert	4	0	4	0	0	0	4	0	4	0	0	0	0	0	0
Keine Angabe	370	181	551	55	27	82	229	93	322	64	21	85	18	2	20

^a^ Geschmacks- und Geruchssinnveränderungen wurden in Phase 1 noch nicht systematisch erfasst und sind deshalb unterschätzt

^b^ Status nach Infektionsschutzgesetz (IfSG): Betreuung oder Tätigkeit nach §23 v. a. in Krankenhäusern und anderen medizinischen Funktionen, nach §33 v. a. in Kitas, Kinderhorten, Schulen, Heimen und Ferienlagern; nach §36 v. a. in Pflegeeinrichtungen, Obdachlosenunterkünften, Einrichtungen für Asylsuchende, sonstigen Massenunterkünften, Justizvollzugsanstalten; nach §42: Tätigkeit v. a. in Fleischindustrie, Küchen von Gaststätten und Gemeinschaftsverpflegung

Die Inzidenzen (jeweils bezogen auf 100.000 Einwohner) variierten in der ersten Phase von 34 (Ostholstein) bis 190 (Pinneberg) und in der zweiten Phase von 15 (Schleswig-Flensburg) bis 114 (Dithmarschen). Die Hospitalisierungsraten rangierten in der ersten Phase zwischen 4,8 % (Lübeck) und 22,9 % (Kiel) und in der zweiten Phase zwischen 2,7 % (Flensburg) und 19,1 % (Plön). Bezüglich der Letalität lag in der ersten Phase das Maximum bei 7,9 % (Stormarn) und in der zweiten Phase bei 2,0 % in Nordfriesland, während aus Ostholstein in diesem Zeitraum kein Todesfall berichtet wurde (Tab. 1).

Während die Geschlechterverteilung insgesamt ausgeglichen war (*n* = 2336 männlich, *n* = 2491 weiblich), überwogen bei den Verstorbenen die Männer (*n* = 97; Letalität 4,2 %) im Vergleich zu den Frauen (*n* = 65; Letalität 2,6 %; Tab. 2). Das durchschnittliche Alter aller infizierten Personen betrug in der ersten Phase 50,7 Jahre und in der zweiten Phase 34,3 Jahre (*p* = 0,0047 im ungepaarten t‑Test). Ebenfalls waren die Altersunterschiede hospitalisierter Personen im Vergleich der beiden Phasen hoch signifikant (*p* = 0,0014 im ungepaarten t‑Test bei einem Mittelwert von 68,4 Jahren in der 1. Phase und 55,9 Jahren in der 2. Phase). Das durchschnittliche Alter der Verstorbenen war 79,2 Jahre und lag bei Frauen mit 83,2 höher als bei Männern mit 76,6 Jahren.

Die häufigsten Symptome waren in beiden Phasen Fieber (37,1 % vs. 22,9 %), Husten (47,7 % vs. 27,5 %) und Allgemeinsymptome (41,1 % vs. 34,6 %). Veränderungen der Geruchs- und Geschmackssinne wurden in der ersten Phase noch nicht systematisch erfasst und sind daher unterschätzt, während sie in der zweiten Phase bei 13,2 % der infizierten Personen auftraten. Die häufigsten Risikokonstellationen für schwere Verläufe von SARS-CoV-2-Infektionen betrafen das Herz-Kreislauf-System (*n* = 565), das Nervensystem (*n* = 205) und die Lungen (*n* = 205). Während der ersten Phase waren 17,2 % aller infizierten Personen asymptomatisch, während der zweiten Phase 38,7 %.

In den Meldedaten werden Fälle auch nach der Zugehörigkeit in Betreuung oder Tätigkeit unterschieden nach §23 IfSG vor allem in Krankenhäusern und anderen medizinischen Funktionen, nach §33 vor allem in Kitas, Kinderhorten, Schulen, Heimen und Ferienlagern, nach §36 vor allem in Pflegeeinrichtungen, Obdachlosenunterkünften, Einrichtungen für Asylsuchende, sonstigen Massenunterkünften, Justizvollzugsanstalten und nach §42 vor allem in der Fleischindustrie und Küchen von Gaststätten und Gemeinschaftsverpflegung. Nach den §§23, 33 bzw. 36 des IfSG betreute Personen waren in insgesamt 107, 250 bzw. 340 Fällen infiziert, von denen 14, 0 bzw. 70 Personen verstarben (Tab. 2). In den Bereichen nach §§23, 33, 36 und 42 tätige Personen waren in 424, 101, 207 bzw. 215 Fällen infiziert. Bezüglich der ausländischen Expositionsorte dominierten in der ersten Phase Österreich und Südeuropa und in der zweiten Phase Südosteuropa und die Türkei. Nur wenige Personen mit ausländischem Expositionsort verstarben an den Folgen der Infektion (*n* = 8).

Im Beobachtungszeitraum wurden 412 Infektionsherde festgestellt, von denen 204 mindestens drei Personen umfassten. In der ersten Phase wurden 250 Herde mit insgesamt 1004 Personen dokumentiert, in der zweiten Phase 162 Herde mit 579 Beteiligten (Tab. 3A). Bei den entsprechenden Infektionsumfeldern dominierten insgesamt 278 Ausbrüche mit 843 Fällen aus dem Setting des privaten Haushalts, der aber viele andere, nicht konkret definierbare Infektionsmöglichkeiten mit umfasst (Tab. 3B). Alten- und Pflegeheime waren mit 24 Ausbrüchen und 243 Fällen bei 34 Verstorbenen hinsichtlich der Ausbruchs- und Fallzahl das zweitwichtigste Setting. 17 übergeordnete Ausbrüche betrafen mehrere Kreise und kreisfreie Städte. Der größte, kreisübergreifende Ausbruch in einem Schlachthof umfasste insgesamt 139 Personen. Außerdem sind unter diesen übergeordneten Ausbrüchen auch ein besonders großer Herd in einem Alten- und Pflegeheim mit 81 infizierten Personen und ein Ausbruch nach einem Spiel der Handballbundesliga enthalten.

**Table 3:** 

A							B				
Ausbruchsgröße^a^	Phase 1		Phase 2			Insgesamt	Infektionsumfeld	Ausbrüche	Fälle	Hospitalisiert	Verstorben
	Fälle	Herde	Fälle	Herde		Fälle					
1‑2	139	255	69	117	208	372	Privater Haushalt	278	843	71	6
3‑4	69	227	56	190	125	417	Alten‑/Pflegeheim	24	243	26	34
5‑6	19	101	22	118	41	219	Arbeitsplatz	24	75	6	1
7‑8	7	52	5	38	12	90	Hotel, Pension, Herberge	19	68	2	0
9‑10	2	19	6	57	8	76	Krankenhaus	17	77	31	3
11-12	2	23	1	11	3	34	Freizeit, Verein, Picknick, Ähnliches	13	44	7	1
13-14	2	27	1	13	3	40	Ambulante Behandlungseinrichtung, Praxis	8	26	3	0
16-17	1	16	1	17	2	33	Schule	6	29	3	0
18-19	3	55	1	18	4	73	Wohnheim (Kinder/Jugend/Studierende)	5	49	13	3
22-25	2	47	0	0	2	47	Flugzeug, Bus	4	30	5	2
27-29	2	56	0	0	2	56	Restaurant, Gaststätte	4	12	0	0
46-80	2	126	0	0	2	126	Seniorentagesstätte	3	51	10	13
*Summe*	*250*	*1004*	*162*	*579*	*412*	*1583*	Rehaeinrichtung	2	16	5	1
Summe > 2	111	749	93	462	204	1211	Flüchtlings‑, Asylbewerberheim	2	15	0	0
–	–	–	–	–	–	–	Verstreut	1	3	0	0
–	–	–	–	–	–	–	Andere/sonstige	1	1	0	0
–	–	–	–	–	–	–	Nicht erhoben	1	1	0	0
–	–	–	–	–	–	–	*Insgesamt*	*412*	*1583*	*182*	*64*

^a^ Anzahl beteiligter Infizierter

Die Auswirkung der COVID-19-Pandemie auf die Gesamtmortalität wurde außerdem vergleichend für Schleswig-Holstein und das gesamte Deutschland auf der Basis der veröffentlichten Daten des Statistischen Bundesamts (Destatis) untersucht. Die verfügbaren Daten des Jahres 2020 wurden ausgewertet und dem Mittelwert der Jahre 2016 bis 2019 gegenübergestellt (nicht dargestellt). Für Deutschland lag ein deutlicher Effekt der Pandemie auf die Gesamtmortalität für die 14.–17. Kalenderwoche vor (30.03.–26.04.2020). Dagegen war für Schleswig-Holstein ein sehr schwacher Effekt nur für die 16. Kalenderwoche nachzuweisen (13.04.–19.04.2020). Im Rahmen der einschränkenden Maßnahmen lösten sich diese Auffälligkeiten jedoch rasch auf. Auch wenn in Schleswig-Holstein eine beträchtliche Letalität der SARS-CoV-2-Infektion beobachtet wurde, wirkte sich diese noch nicht wesentlich auf die Gesamtmortalität aus. Damit kann weitestgehend ausgeschlossen werden, dass im Untersuchungszeitraum ein breiter, unerkannter Eintrag in die Allgemeinbevölkerung vorlag.

## Fazit

Die Coronaviruspandemie verlief in Schleswig-Holstein im ersten Halbjahreszeitraum (März bis September 2020) im Vergleich zum Bundesgebiet relativ günstig mit einer der niedrigsten Landesinzidenzen. Für die Auswertung wurden die Meldedaten der ersten Phase von März bis Mai mit der zweiten Phase von Juni bis September verglichen. Die wesentlichen Unterschiede zwischen den epidemischen Phasen bestanden hinsichtlich der Hospitalisierung und Letalität, der Altersverteilung und der Expositionsländer. In der ersten Phase von März bis Mai 2020 dominierten schwere Erkrankungen älterer Personen mit hohen Raten der Hospitalisierung und Sterblichkeit. In der zweiten Phase von Juni bis September 2020 dominierten jüngere Personen mit niedrigeren Hospitalisierungs- und Sterberaten und mit deutlicher Reiseaktivität. Die meisten Ausbrüche wurden für private Haushalte dokumentiert. Die Pandemie hatte im Beobachtungszeitraum kaum Auswirkungen auf die Gesamtmortalität in Schleswig-Holstein.
